# Sex-specific differences in survival after out-of-hospital cardiac arrest: a nationwide, population-based observational study

**DOI:** 10.1186/s13054-019-2547-x

**Published:** 2019-07-25

**Authors:** Yoshikazu Goto, Akira Funada, Tetsuo Maeda, Hirofumi Okada, Yumiko Goto

**Affiliations:** 1 Department of Emergency and Critical Care Medicine, Kanazawa University Hospital, Takaramachi 13-1, Kanazawa, 920-8640 Japan; 2 Department of Cardiology, Yawata Medical Center, Yawata I 12-7, Komatsu, 923-8551 Japan

**Keywords:** Cardiopulmonary resuscitation, Out-of-hospital cardiac arrest, Sex difference, Epidemiology

## Abstract

**Background:**

It remains unclear whether men have more favorable survival outcomes after out-of-hospital cardiac arrest (OHCA) than women.

**Methods:**

We reviewed a total of 386,535 patients aged ≥ 18 years with OHCA who were included in the Japanese registry from 2013 to 2016. The study endpoints were the rates of 1-month survival and neurologically intact survival (Cerebral Performance Category Scale score = 1 or 2). Based on age, the reviewed patients were categorized into the following eight groups: < 30, 30–39, 40–49, 50–59, 60–69, 70–79, 80–89, and ≥ 90 years. The survival outcomes in men and women were compared using hierarchical propensity score matching.

**Results:**

The crude survival rate was significantly higher in men than in women in five groups: 30–39, 40–49, 50–59, 60–69, and 70–79 years (all *P* < 0.001). Similarly, the crude neurologically intact survival rate was significantly higher in men than in women in seven groups: < 30, 30–39, 40–49, 50–59, 60–69, 70–79, and 80–89 years (all *P* < 0.005). However, multivariate logistic regression analysis of each group revealed no significant sex-specific differences in 1-month survival outcomes (all *P* > 0.02). Moreover, after hierarchical propensity score matching, the survival outcomes did not significantly differ between both sexes (all *P* > 0.05).

**Conclusions:**

No significant sex-specific differences were found in the rates of 1-month survival and neurologically intact survival after OHCA.

**Electronic supplementary material:**

The online version of this article (10.1186/s13054-019-2547-x) contains supplementary material, which is available to authorized users.

## Background

Location of arrest, younger age, presence of witnesses, bystander cardiopulmonary resuscitation (CPR), initial shockable rhythm, early defibrillation, short CPR duration, short emergency medical service (EMS) response time, and prompt coronary angiography with percutaneous coronary intervention if indicated are factors reportedly associated with survival in patients with out-of-hospital cardiac arrest (OHCA) [1–5]. In terms of sex differences related to patient outcomes after OHCA, some studies reported that compared with men of the same age, women of childbearing age were independently associated with improved survival [6–14]; this survival advantage might be attributable to the protective anti-apoptotic, anti-inflammatory, and mitochondria-stabilizing activities of estrogen [6, 12, 15]. Other studies failed to demonstrate such an advantage but instead reported equal or worse survival rates or lower quality of life among female OHCA survivors [16–21]. Inconsistent results reported by previous observational studies may be due to differences in the study populations, EMS systems, and patient risk factors. A previous study reported differences in the baseline characteristics of women and men with OHCA. Compared with the women, the men were reportedly younger, and arrest was more frequently witnessed in the men than in the women; bystander CPR, cardiac etiology, and initial shockable rhythm were also more frequent in the men than in the women [12]. Rigorous statistical analysis is needed to confirm the differences in outcomes after OHCA between women and men. Therefore, in the present study, hierarchical propensity score matching was used to analyze and compare the outcomes after OHCA between age-stratified women and men who were included in a Japanese registry.

## Methods

### Study design and setting

This nationwide, population-based observational study included 386,535 adult patients aged ≥ 18 years with OHCA. In all these patients, resuscitation was attempted by EMS personnel in Japan between January 1, 2013, and December 31, 2016. In Japan, nearly 127 million individuals reside in an area of approximately 380,000 km^2^. Further, approximately two thirds of Japan comprises uninhabited mountainous terrain [22]. The Fire and Disaster Management Agency (FDMA) of Japan supervises a nationwide EMS system, whereas local fire stations operate local EMS systems. In 2017, Japan had 732 fire departments and 5140 ambulance teams [23]. During the study period, all EMS personnel performed CPR following the Japanese CPR guidelines and attempted resuscitation by using automated external defibrillators, inserting airway adjuncts and peripheral intravenous catheters, and administering Ringer’s lactate solution [23–25]. Only specially trained emergency life-saving technicians are permitted to insert tracheal tubes and administer intravenous epinephrine after receiving online instructions from a physician [23]. Except in special situations, such as decapitation, incineration, decomposition, rigor mortis, and dependent cyanosis, EMS personnel in Japan are legally prohibited from terminating resuscitation in the field. Most patients with OHCA were given CPR by EMS personnel before transport to a hospital.

### Data collection and quality control

In 2005, the FDMA launched an ongoing prospective population-based observational study including all patients with OHCA in Japan who received resuscitation by EMS personnel [23]. EMS personnel and the physician in charge at each center recorded data from the patients using an Utstein-style recommended guideline template [26, 27]. The data were transferred to individual local fire stations and subsequently integrated into the registry on the FDMA database server. The database application automatically checked the patient data for consistency, which was again verified by the FDMA. The data were transferred to and stored in a nationwide database that was developed by the FDMA for public use. The FDMA granted us permission to access the anonymized data for this study.

The characteristics included in the dataset were as follows: patient sex and age, etiology of arrest, initially identified cardiac rhythm, presence and relation of bystander witnesses (e.g., family member, a layperson other than family, or EMS personnel), maneuver of bystander CPR, time of collapse, receipt of emergency calls, time of vehicle arrival at the scene and EMS initiation of CPR, 1-month survival, and neurologically intact survival. The etiology of arrest was presumed to be cardiac unless suitable evidence suggested a nonmedical cause (e.g., trauma, accidental hypothermia, hanging, drowning, drug overdose or poisoning, or asphyxia) or another noncardiac cause, such as respiratory or cerebrovascular disease or malignant tumors. The physicians in charge determined the etiology of arrest. Neurological outcomes were defined using the Cerebral Performance Category (CPC) Scale scores (1: good cerebral performance, 2: moderate cerebral disability, 3: severe cerebral disability, 4: coma or vegetative state, 5: death) [26]. The CPC scores were determined by the physician in charge.

### Study endpoints

The primary study endpoint was neurologically intact survival (CPC Scale score = 1 or 2 at 1 month). The secondary endpoint was 1-month survival after OHCA.

### Statistical analysis

Continuous variables were either expressed as medians and 25th–75th percentiles or as means and standard deviation. Categorical variables were expressed as numbers and percentages. Effect size and variability were reported as odds ratios (ORs) with 95% confidence intervals (CIs). To determine the differences in 1-month outcomes after OHCA based on sex, the patients were divided into the following eight groups: < 30, 30–39, 40–49, 50–59, 60–69, 70–79, 80–89, and ≥ 90 years. The Kruskal–Wallis and Dunn’s post hoc tests were used to compare continuous variables. Chi-square test was used to compare categorical variables, and univariate logistic regression analysis was performed to compare the characteristics and outcomes between men and women. Multivariate logistic regression analyses were performed after adjusting for the differences in patient baseline characteristics for all unmatched patients and each age group for both matched and unmatched patients. Hierarchical propensity score matching was used to adjust for covariates when comparing the outcomes in men and women. Potential prehospital confounders in the analytic model were selected based on biological plausibility and data reported in previous studies. Multivariate logistic regression analysis in both matched and unmatched patients included the following 12 prehospital variables: calendar year (as a categorical variable), Japan geographic regions (rural or urban area), age (as a continuous variable), sex (men or women), presence of a witness (no witness, witnessed by family member, or nonfamily member), initial cardiac rhythm (shockable or nonshockable), cause of arrest (presumed cardiac cause or noncardiac cause), bystander CPR (yes or no), use of advanced airway management (yes or no), epinephrine administration (yes or no), EMS response time (as a continuous variable), and duration between the call to EMS and hospital arrival (as a continuous variable). Hierarchical propensity score matching analyses of the eight groups were performed using a logistic regression model that included the above-mentioned 12 variables (Additional file 1: Table S1–S8). One-to-one nearest neighbor matching was performed between men and women without replacement using a caliper width of 0.20 times of the standard deviation of the logit of the propensity score [28]. Before analyzing the outcomes, the success of the propensity matching procedure was determined by comparing the distributions of the patient characteristics in the matched sample by calculating an absolute standardized difference [29]. An absolute standardized difference of ≥ 0.1 indicated a significant difference between the sexes [30]. The outcomes of the men and women in each age group were compared before propensity matching with either the chi-square test or Fisher’s exact test and after propensity matching with the McNemar’s test. All data were analyzed using JMP statistical package software version 14-Pro (SAS Institute Inc.; Cary, NC, USA). All the reported tests were two-tailed, and *P* < 0.005 was considered statistically significant [31, 32].

## Results

**Fig. 1:**
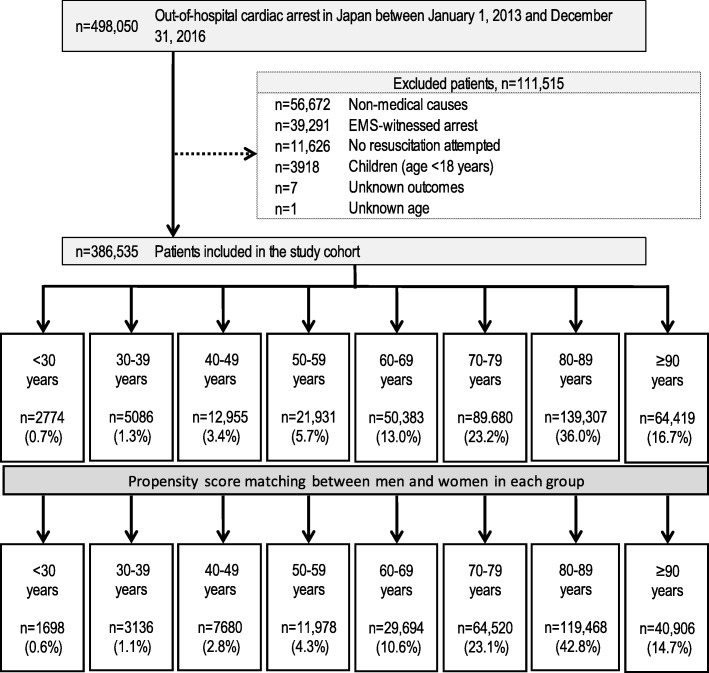
Study inclusion flowchart. EMS emergency medical services

From 2013 to 2016, in Japan, the details of attempted resuscitation for 498,050 patients with OHCA were documented in the FDMA database. Figure 1 indicates the inclusion and exclusion criteria of the present study. Patients with nonmedical causes of OHCA (e.g., trauma, accidental hypothermia, hanging, drowning, drug overdose or poisoning, or asphyxia) and EMS-witnessed arrest, those without resuscitation attempted by EMS personnel, those aged < 18 years, and those with unknown outcomes or age, were excluded. A group of 386,535 patients (77.6% of those in the registry) met the inclusion criteria and were hence included in this study. Patient matching was achieved for 72.2% (279,080 of 386,535) of the patients [64.3% (139,540 of 217,173) for men; 82.4% (139,540 of 169,362) for women]. Propensity matching considerably improved the absolute standardized differences in each age group (Additional file 1: Table S1–S8).

Table 1 presents the baseline characteristics of the women and men included in this study. Compared with the women, the included men were younger, less likely to experience OHCA in rural areas, and more likely to have a presumed cardiac origin and initial shockable rhythm; they were also more likely to be witnessed by a family member and receive advanced airway maintenance and epinephrine. In contrast, more women than men received bystander CPR following the instructions given by an EMS dispatcher. The overall crude 1-month survival outcomes were significantly better in men than in women. Multivariate logistic regression analysis in unmatched patients (Table 2) revealed that compared with women, men were independently associated with increased odds of achieving 1-month favorable outcomes (survival, adjusted OR = 1.07; 95% CI 1.03–1.11; CPC 1–2, adjusted OR = 1.22; 95% CI 1.16–1.29).

**Table 1:** Baseline characteristics of the participants by sex

Characteristic	Men	Women	*P* value
	*n* = 217,173 (56.2%)	*n* = 169,362 (43.8%)	
Year			0.95
2013	53,459 (24.6)	41,693 (24.6)	
2014	55,123 (25.4)	42,925 (25.4)	
2015	54,061 (24.9)	42,294 (24.9)	
2016	54,530 (25.1)	42,450 (25.1)	
Geographic Japanese regions			
Rural area*	54,143 (24.9)	43,512 (25.7)	< 0.001
Age, years			< 0.001
Mean (SD)	74.3 (14.1)	80.3 (13.2)	
Median (25–75%)	77 (67–85)	84 (75–90)	
Etiology of cardiac arrest			
Presumed cardiac cause	152,531 (70.2)	116,994 (69.1)	< 0.001
Initial shockable rhythm	23,159 (10.7)	7248 (4.3)	< 0.001
Bystander witness status			< 0.001
No witness	129,709 (59.7)	107,825 (63.7)	
Family member	57,967 (26.7)	35,544 (21.0)	
Nonfamily member	29,497 (13.6)	25,993 (15.3)	
Dispatcher CPR instruction			
Offered	126,685 (58.3)	105,020 (62.0)	< 0.001
Bystander CPR	107,618 (49.6)	95,609 (56.4)	< 0.001
Use of advanced airway management	94,481 (43.5)	69,107 (40.8)	< 0.001
Epinephrine administration	44,648 (20.6)	28,120 (16.6)	< 0.001
Call-to-response time, min			< 0.001
Mean (SD)	9.2 (3.9)	9.1 (3.6)	
Median (25–75%)	8 (7–11)	8 (7–11)	
Call-to-hospital arrival time, min			< 0.001
Mean (SD)	31.8 (10.4)	31.5 (10.1)	
Median (25–75%)	31 (25–38)	31 (25–38)	
1-month outcome			
Survival	12,373 (5.7)	5,561 (3.3)	< 0.001
CPC 1 or 2	6936 (3.2)	2,327 (1.4)	< 0.001

Values are reported as *n* (%) unless indicated otherwise. *CPC*, Cerebral Performance Category; *CPR*, cardiopulmonary resuscitation; *SD*, standard deviation

*The rural area is constituted 19 prefectures with population of less than 200 inhabitants per km^2^

**Table 2:** Adjusted odds ratios of prehospital variables for 1-month outcomes in unmatched patients (*n* = 386,535)

Variables	Adjusted OR (95% CI)	
	1-month survival	1-month CPC 1 or 2
Year		
2014 (vs. 2013)	1.04 (0.99–1.09)	1.03 (0.96–1.10)
2015 (vs. 2013)	1.10 (1.05–1.15)	1.12 (1.05–1.20)
2016 (vs. 2013)	1.15 (1.10–1.21)	1.23 (1.15–1.31)
Geographic Japanese regions		
Rural area* (vs. urban area)	1.03 (0.99–1.07)	1.02 (0.96–1.07)
Age^†^	0.97 (0.97–0.97)	0.96 (0.96–0.97)
Men (vs. women)	1.07 (1.03–1.11)	1.22 (1.16–1.29)
Witnessed arrest (vs. unwitnessed arrest)		
Family member	3.96 (3.80–4.13)	4.16 (3.91–4.42)
Nonfamily member	4.86 (4.65–5.08)	5.49 (5.16–5.85)
Initial shockable rhythm (vs. initial nonshockable rhythm)	7.31 (7.03–7.60)	9.17 (8.70–9.65)
Presumed cardiac cause (vs. noncardiac causes)	0.79 (0.76–0.82)	1.37 (1.29–1.46)
Bystander CPR (vs. no bystander CPR)	1.16 (1.12–1.19)	1.41 (1.34–1.48)
Use of advanced airway management (vs. no use of airway management)	0.69 (0.67–0.72)	0.42 (0.40–0.44)
Epinephrine administration (vs. no use of epinephrine)	0.81 (0.77–0.84)	0.40 (0.37–0.43)
Call-to-response time^†^	0.91 (0.90–0.91)	0.90 (0.89–0.90)
Call-to-hospital arrival time^†^	0.99 (0.99–1.00)	1.00 (0.99–1.00)

*CI*, confidence interval; *CPC*, Cerebral Performance Category; *CPR*, cardiopulmonary resuscitation; *OR*, odds ratio

*The rural area is constituted 19 prefectures with population of less than 200 inhabitants per km^2^

^†^Adjusted odds ratios are reported for 1-year or 1-min increments

**Fig. 2:**
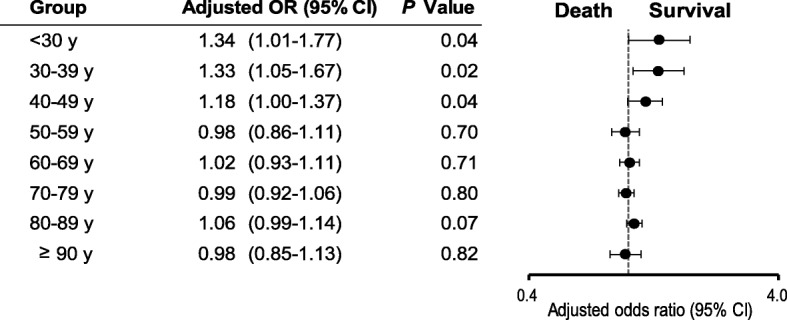
Adjusted OR of women compared with men for 1-month survival in unmatched patients by age. CI confidence interval, OR odds ratio

**Fig. 3:**
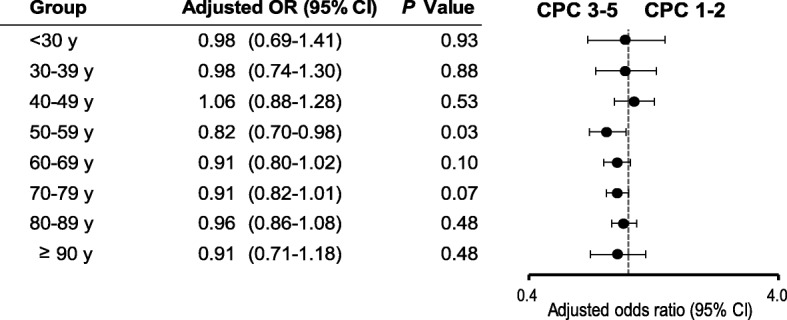
Adjusted OR of women compared with men for 1-month CPC 1–2 in unmatched patients by age. CI confidence interval, CPC Cerebral Performance Category scale, OR odds ratio

**Fig. 4:**
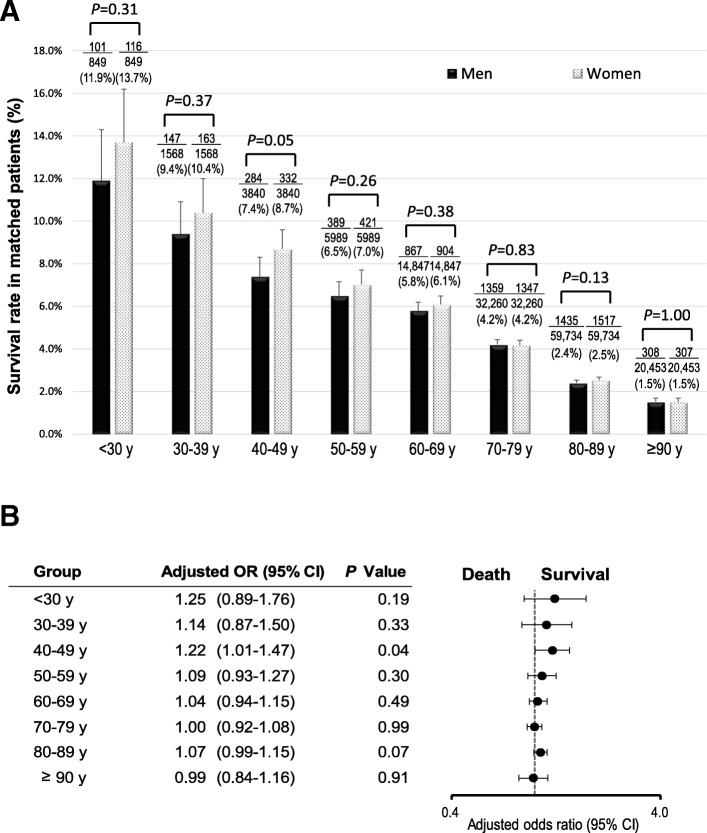
One-month survival rate in matched patients by age. **a** Survival rate. **b** Adjusted OR of women compared with men for 1-month survival. CI confidence interval, OR odds ratio

**Fig. 5:**
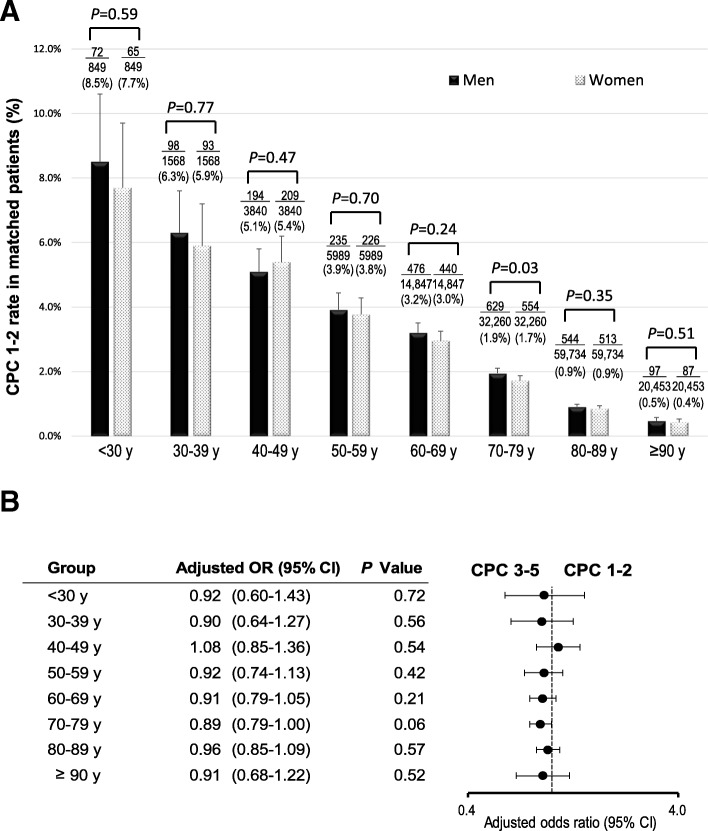
One-month CPC 1–2 rate in matched patients by age. **a** CPC 1–2 rate. **b** Adjusted OR of women compared with men for 1-month CPC 1–2. CI confidence interval, CPC Cerebral Performance Category scale, OR odds ratio

The crude 1-month survival outcomes in unmatched patients in each age group are presented in Additional file 2: Figure S1. Survival decreased with increasing age in both women and men (all *P* for trend < 0.001). The crude 1-month survival rate was significantly higher in men than in women in five groups: 30–39, 40–49, 50–59, 60–69, and 70–79 years. Multivariate logistic regression analysis revealed no significant sex-specific differences in the odds of 1-month survival (Fig. 2). The crude 1-month CPC 1–2 in the unmatched patients in each age group is shown in Additional file 3: Figure S2. The crude 1-month CPC 1–2 gradually reduced with increasing age in both women and men (all *P* for trend < 0.001). The crude 1-month CPC 1–2 were significantly higher in men than in women in seven groups: < 30, 30–39, 40–49, 50–59, 60–69, 70–79, and 80–89 years. No significant differences in the 1-month CPC 1–2 were found between women and men after adjusting for confounders (Fig. 3). Figures 4 and 5 present the 1-month survival outcomes of matched patients in each age group. There were no significant sex-specific differences in the adjusted odds of survival and CPC 1–2 in any group.

## Discussion

Hierarchical propensity score matching did not indicate any differences in the 1-month survival and neurologically intact survival between men and women in any of the age groups analyzed in this nationwide, population-based observational study. This is the first large cohort study to clearly demonstrate the absence of differences in clinically meaningful survival between men and women after OHCA.

Hormonal effect on survival outcomes after cardiac arrest remains controversial: estrogen mediates hormonal responses to ischemia–reperfusion injury in women of childbearing age [33–37], whereas androgen mediates negative effects on the cardiovascular system in young men [38–40]. Previous studies have revealed that apart from reproductive roles, sex hormones possess cardioprotective and neuroprotective functions [41, 42]. Estrogen has previously been reported to have cardioprotective and neuroprotective effects after cardiac arrest [35–37]. Although the cardioprotective effects of estrogen are widely appreciated, little is known about the effects of progesterone [42]. Testosterone is associated with various adverse events in young men, including coronary plaque formation, platelet aggregation, inflammation, heart failure, and coronary artery disease [38–40]. Increased testosterone levels in men have been associated with decreased sudden cardiac arrest events; further, increased estradiol levels in both sexes have been associated with increased sudden cardiac arrest events [43]. Nevertheless, the impact of testosterone on the cardiovascular system remains controversial [42]. However, in the present study, there were no significant sex-specific differences in the 1-month survival outcomes in each age group after adjustment for prehospital confounders. These results possibly reflect estrogen-related effects on women and testosterone-related effects on men. Regarding the neuroprotective effects of sex hormones, estrogen reportedly slows down the progression of brain injury and diminishes the extent of cell death by suppressing apoptotic pathways [44]. In agreement with that report, another study demonstrated that brain edema after intracerebral hemorrhage-induced injury was reportedly less severe in female than in male rats [45]. The neuroprotective properties of progesterone following cerebral ischemia, which are mediated by a reduction in edema, are most likely related to the suppression of interleukin-1β production [46]. Testosterone also possesses neuroprotective properties that result from the activation of androgen pathways as well as antioxidant and anti-apoptotic activities. The neuroprotective effects of sex hormones may thus partially explain the lack of significant differences in the neurologically intact survival outcomes between women and men in this study [41]. Other factors that may have contributed to the sex-specific differences in survival outcomes after OHCA include sex-specific anatomical differences of chest wall compliance [47], coronary risk factors [48], genomic variation [49], and care process [50]. The statistical analysis did not adjust for those factors as confounding variables because of the lack of data in the registry.

A recent analysis by Benjamin et al. proposed changing the significance level from *P* < 0.05 to *P* < 0.005 to avoid a high rate of false-positive results, even in the absence of other reporting problems [32]. Thus, the lack of reproducibility of sex differences and outcomes after OHCA in previous studies might be related to the statistical analysis. In this study, *P* < 0.005 was considered statistically significant. Our findings that female patients with OHCA were older, were more likely to present with nonshockable rhythms, had more unwitnessed arrests, and received bystander CPR more frequently than male patients are consistent with the findings of some [6, 12, 14, 20] but not all previous studies [8–11, 16]. Our findings showed that women of childbearing age had no survival advantage over age-matched men after OHCA, which differs from previously reported results in Japan [6, 7, 14]. For example, Kitamura et al. reported that from 1998 to 2007 in Osaka, female patients aged > 13 years had higher 1-month survival rates and that those aged 13–49 years had higher 1-month neurologically intact survival rates after OHCA compared with male patients of the same age [6]. Using the 2005–2007 Utstein data, Akahane et al. reported that female patients aged 30–79 years with OHCA with an initial shockable rhythm had improved 1-month survival and that those aged 40–59 years had better 1-month neurologically intact survival than male patients of the same age [7]. Taken together, these findings indicate that the survival rates were not solely attributable to the effects of estrogen. In addition, we used hierarchical propensity score matching analysis within each age group to adjust for confounding factors; this was not followed by previous studies [6, 7]. Differences in the results reported in the above studies may also reflect improvements in the management OHCA after the 2010 and 2015 updates of the international CPR guidelines. Previous studies from Korea, Australia, and New Zealand reported no sex-specific differences in survival outcomes after OHCA, which is consistent with our results [16, 17, 51]. Importantly, our data further showed the relationship between sex and neurologically intact survival after cardiac arrest in which sex was not associated with clinically meaningful survival.

The present study has some limitations. First, the study analyzed data collected from a large national population by standard procedures; however, because of the retrospective observational design, we could not exclude uncontrolled confounders. For example, patient estrogen, progesterone, and testosterone levels were not available. Sex difference was a surrogate for both sex-related (sex hormone levels, autonomic modulation, and electrophysiological properties) and gender-specific (socioeconomic, environmental, educational, and community) factors that could not be adjusted for. A prospective study of patients with OHCA including sex hormone assays would be needed to clarify the relationship between sex hormone levels and outcomes. The present study also lacked data on pre-existing comorbidities, the location of arrest, the quality of bystander- and EMS-initiated CPR, and the in-hospital treatments. Even in propensity score matching analysis, we cannot exclude numerous unknown confounding factors that may mislead the sex-specific differences in the outcomes after OHCA. Other limitations are common to epidemiological studies, including ascertainment bias and lack of data integrity and validity. The relevance of our results to other communities with different emergency care systems and protocols is not known; similar studies in other countries would help validate our results.

## Conclusions

The analyses of the Japanese nationwide registry revealed no significant sex-specific differences in the 1-month survival and neurologically intact survival rates after OHCA.

## Additional files

Additional file 1: **Table S1.** Baseline characteristics of unmatched and matched patients aged < 30 years. **Table S2.** Baseline characteristics of unmatched and matched patients aged 30–39 years. **Table S3.** Baseline characteristics of unmatched and matched patients aged 40–49 years. **Table S4.** Baseline characteristics of unmatched and matched patients aged 50–59 years. **Table S5.** Baseline characteristics of unmatched and matched patients aged 60–69 years. **Table S6.** Baseline characteristics of unmatched and matched patients aged 70–79 years. **Table S7.** Baseline characteristics of unmatched and matched patients aged 80–89 years. **Table S8.** Baseline characteristics of unmatched and matched patients aged ≥ 90 years. (DOCX 135 kb)

Additional file 2: **Figure S1.** One-month crude survival rate in unmatched patients by age *P < 0.001. The trends of both sexes for increasing age groups were significant (all *P* for trend < 0.001). (PPTX 503 kb)

Additional file 3: **Figure S2.** One-month crude CPC 1–2 rate in unmatched patients by age CPC: Cerebral Performance Category scale. *P < 0.001. ^†^*P* < 0.005. The trends of both sexes for increasing age groups were significant (all *P* for trend < 0.001). (PPTX 534 kb)

## Data Availability

The datasets generated during and/or analyzed during the current study are not publicly available because of the Fire and Disaster Management Agency (FDMA) regulations but are available from the corresponding author on reasonable request.

## Abbreviations

CI: Confidence interval
CPC: Cerebral Performance Category
CPR: Cardiopulmonary resuscitation
EMS: Emergency medical service
FDMA: Fire and Disaster Management Agency
OHCA: Out-of-hospital cardiac arrest
OR: Odds ratio
