# Supplementary material for: Sex-specific differences in survival after out-of-hospital cardiac arrest: a nationwide, population-based observational study
Source: Crit Care. 2019 Jul 25;23:263. doi: 10.1186/s13054-019-2547-x (PMC6659261; doi:10.1186/s13054-019-2547-x)
Supplement: Supplementary file 1 — Table S1. Baseline characteristics of unmatched and matched patients aged < 30 years. Table S2. Baseline characteristics of unmatched and matched patients aged 30–39 years. Table S3. Baseline characteristics of unmatched and matched patients aged 40–49 years. Table S4. Baseline characteristics of unmatched and matched patients aged 50–59 years. Table S5. Baseline characteristics of unmatched and matched patients aged 60–69 years. Table S6. Baseline characteristics of unmatched and matched patients aged 70–79 years. Table S7. Baseline characteristics of unmatched and matched patients aged 80–89 years. Table S8. Baseline characteristics of unmatched and matched patients aged ≥ 90 years. (DOCX 135 kb) [file 13054_2019_2547_MOESM1_ESM.docx]

**Additional file 1**

**Table S1.** Baseline characteristics of unmatched and matched patients aged <30 years

| Characteristic | | | Pre-propensity-matched patients | | | | |  | Post-propensity-matched patients | | | | | | | | | |  |
| --- | --- | --- | --- | --- | --- | --- | --- | --- | --- | --- | --- | --- | --- | --- | --- | --- | --- | --- | --- |
|  | | | Men | | Women | | ASD* |  | Men | | | | Women | | | | ASD* | |  |
|  |  |  | (n=1922) | | (n=852) | |  |  | (n=849) | | | | (n=849) | | | |  |  |  |
| Year | | |  |  |  |  |  |  |  | |  | |  | |  | |  | |  |
|  | 2013 | | 516 | (26.9) | 207 | (24.3) | 0.06 |  | 209 | | (24.6) | | 206 | | (24.3) | | 0.01 | |  |
|  | 2014 | | 463 | (24.1) | 221 | (25.9) | 0.04 |  | 220 | | (25.9) | | 220 | | (25.9) | | 0 | |  |
|  | 2015 | | 487 | (25.3) | 204 | (23.9) | 0.03 |  | 203 | | (23.9) | | 204 | | (24.0) | | <0.01 | |  |
|  | 2016 | | 456 | (23.7) | 220 | (25.8) | 0.05 |  | 217 | | (25.6) | | 219 | | (25.8) | | 0.01 | |  |
| Geographic Japanese regions | | |  |  |  |  |  |  |  | |  | |  | |  | |  | |  |
|  | Rural area† | | 461 | (24.0) | 219 | (25.7) | 0.04 |  | | 234 | | (27.6) | | 218 | | (25.7) | | 0.04 | |
| Age, y, mean (SD) | | | 24.0 | (3.4) | 24.1 | (3.4) | 0.02 |  | | 24.1 | | (3.4) | | 24.1 | | (3.5) | | <0.01 | |
| Etiology of cardiac arrest | | |  |  |  |  |  |  | |  | |  | |  | |  | |  | |
|  | Presumed cardiac origin | | 1090 | (56.7) | 420 | (49.3) | 0.15 |  | | 415 | | (48.9) | | 418 | | (49.2) | | 0.01 | |
| Initial cardiac rhythm | | |  |  |  |  |  |  | |  | |  | |  | |  | |  | |
|  | Shockable | | 402 | (20.9) | 100 | (11.7) | 0.25 |  | | 99 | | (11.7) | | 100 | | (11.8) | | <0.01 | |
| Bystander witness status | | |  |  |  |  |  |  | |  | |  | |  | |  | |  | |
|  | No witness | | 1178 | (61.3) | 572 | (67.1) | 0.12 |  | | 567 | | (66.8) | | 570 | | (67.1) | | 0.01 | |
|  | Witnessed by family member | | 342 | (17.8) | 178 | (20.9) | 0.08 |  | | 185 | | (21.8) | | 177 | | (20.9) | | 0.02 | |
|  | Witnessed by nonfamily member | | 402 | (20.9) | 102 | (12.0) | 0.24 |  | | 97 | | (11.4) | | 102 | | (12.0) | | 0.02 | |
| Dispatcher CPR instruction | | |  |  |  |  |  |  |  | |  | |  | |  | |  | |  |
|  | Offered | | 1070 | (55.7) | 508 | (59.6) | 0.08 |  | 495 | | (58.3) | | 507 | | (59.7) | | 0.03 | |  |
| Bystander intervention | | |  |  |  |  |  |  |  | |  | |  | |  | |  | |  |
|  | Bystander defibrillation | | 2 | (0.1) | 1 | (0.1) | <0.01 |  | 0 | | (0.0) | | 1 | | (0.1) | | 0.05 | |  |
|  | Bystander conventional CPR | | 194 | (10.1) | 76 | (8.9) | 0.04 |  | 70 | | (8.2) | | 75 | | (8.8) | | 0.02 | |  |
|  | Bystander compression-only CPR | | 912 | (47.5) | 402 | (47.2) | 0.01 |  | 402 | | (47.4) | | 402 | | (47.4) | | 0 | |  |
|  | Bystander rescue breathing-only CPR | | 11 | (0.6) | 7 | (0.8) | 0.03 |  | 4 | | (0.5) | | 7 | | (0.8) | | 0.04 | |  |
|  | No bystander intervention | | 803 | (41.8) | 366 | (43.0) | 0.02 |  | 373 | | (43.9) | | 364 | | (42.9) | | 0.02 | |  |
| Use of advanced airway management | | | 633 | (32.9) | 288 | (33.8) | 0.02 |  | 290 | | (34.2) | | 286 | | (33.7) | | 0.01 | |  |
| Epinephrine administration | | | 336 | (17.5) | 103 | (12.1) | 0.15 |  | 98 | | (11.5) | | 103 | | (12.1) | | 0.02 | |  |
| Call-to-response time, min, mean (SD) | | | 9.51 | (4.7) | 9.04 | (3.7) | 0.11 |  | 9.22 | | (4.2) | | 9.04 | | (3.7) | | 0.04 | |  |
| Call-to-hospital arrival time, min, mean (SD) | | | 31.4 | (10.7) | 31.6 | (10.4) | 0.01 |  | 31.2 | | (10.4) | | 31.5 | | (10.4) | | 0.03 | |  |
| Values are reported as n (%) unless indicated otherwise. ASD, absolute standardized difference; CPR, cardiopulmonary resuscitation; SD, standard deviation. | | | | | | | | | | | | | | | | | | |  |
| *An ASD of equal or more than 0.1 was considered to indicate a substantial imbalance between the two groups. | | | | | | | | | | | | | | | | | | |  |
| †The rural area is constituted 19 prefectures with population of less than 200 inhabitants per km^2^. | | | | | | | | | | | | | | | | | | |  |

**Table S2.** Baseline characteristics of unmatched and matched patients aged 30–39 years

| Characteristic | | | Pre-propensity-matched patients | | | | |  | Post-propensity-matched patients | | | | |
| --- | --- | --- | --- | --- | --- | --- | --- | --- | --- | --- | --- | --- | --- |
|  | | | Men | | Women | | ASD* |  | Men | | Women | | ASD* |
|  |  |  | (n=3513) | | (n=1573) | |  |  | (n=1568) | | (n=1568) | |  |
| Year | | |  |  |  |  |  |  |  |  |  |  |  |
|  | 2013 | | 892 | (25.4) | 395 | (25.1) | 0.01 |  | 388 | (24.7) | 393 | (25.1) | 0.01 |
|  | 2014 | | 906 | (25.8) | 420 | (26.7) | 0.02 |  | 423 | (27.0) | 419 | (26.7) | 0.01 |
|  | 2015 | | 868 | (24.7) | 392 | (24.9) | <0.01 |  | 397 | (25.3) | 391 | (24.9) | 0.01 |
|  | 2016 | | 847 | (24.1) | 366 | (23.3) | 0.02 |  | 360 | (23.0) | 365 | (23.3) | 0.01 |
| Geographic Japanese regions | | |  |  |  |  |  |  |  |  |  |  |  |
|  | Rural area† | | 837 | (23.8) | 352 | (22.4) | 0.03 |  | 344 | (21.9) | 352 | (22.5) | 0.01 |
| Age, y, mean (SD) | | | 35.3 | (2.8) | 35.3 | (2.8) | 0.03 |  | 35.3 | (2.8) | 35.3 | (2.8) | 0.03 |
| Etiology of cardiac arrest | | |  |  |  |  |  |  |  |  |  |  |  |
|  | Presumed cardiac origin | | 2221 | (63.2) | 786 | (50.0) | 0.27 |  | 783 | (49.9) | 783 | (49.9) | 0 |
| Initial cardiac rhythm | | |  |  |  |  |  |  |  |  |  |  |  |
|  | Shockable | | 857 | (24.4) | 170 | (10.8) | 0.36 |  | 168 | (10.7) | 169 | (10.8) | <0.01 |
| Bystander witness status | | |  |  |  |  |  |  |  |  |  |  |  |
|  | No witness | | 2059 | (58.6) | 1048 | (66.6) | 0.17 |  | 1065 | (67.9) | 1046 | (66.7) | 0.03 |
|  | Witnessed by family member | | 801 | (22.8) | 361 | (23.0) | <0.01 |  | 339 | (21.6) | 358 | (22.8) | 0.03 |
|  | Witnessed by nonfamily member | | 653 | (18.6) | 164 | (10.4) | 0.23 |  | 164 | (10.5) | 164 | (10.5) | 0 |
| Dispatcher CPR instruction | | |  |  |  |  |  |  |  |  |  |  |  |
|  | Offered | | 1936 | (55.1) | 923 | (58.7) | 0.07 |  | 920 | (58.7) | 921 | (58.7) | <0.01 |
| Bystander intervention | | |  |  |  |  |  |  |  |  |  |  |  |
|  | Bystander defibrillation | | 1 | (0.03) | 1 | (0.06) | 0.02 |  | 0 | 0 | 0 | 0 | NA |
|  | Bystander conventional CPR | | 267 | (7.6) | 129 | (8.2) | 0.02 |  | 130 | (8.3) | 129 | (8.2) | <0.01 |
|  | Bystander compression-only CPR | | 1545 | (44.0) | 711 | (45.2) | 0.02 |  | 719 | (45.9) | 710 | (45.3) | 0.01 |
|  | Bystander rescue breathing-only CPR | | 13 | (0.4) | 3 | (0.2) | 0.03 |  | 4 | (0.3) | 3 | (0.2) | 0.01 |
|  | No bystander intervention | | 1687 | (48.0) | 729 | (46.3) | 0.03 |  | 715 | (45.6) | 726 | (46.3) | 0.01 |
| Use of advanced airway management | | | 1414 | (40.3) | 555 | (35.3) | 0.10 |  | 548 | (35.0) | 554 | (35.3) | 0.01 |
| Epinephrine administration | | | 771 | (22.0) | 232 | (14.8) | 0.19 |  | 221 | (14.1) | 232 | (14.8) | 0.02 |
| Call-to-response time, min, mean (SD) | | | 9.36 | (4.4) | 9.19 | (3.4) | 0.04 |  | 9.22 | (3.8) | 9.19 | (3.4) | 0.01 |
| Call-to-hospital arrival time, min, mean (SD) | | | 32.3 | (10.5) | 32.2 | (10.2) | 0.01 |  | 32.1 | (10.3) | 32.2 | (10.2) | 0.01 |
| Values are reported as n (%) unless indicated otherwise. ASD, absolute standardized difference; CPR, cardiopulmonary resuscitation; NA, not available; SD, standard deviation. | | | | | | | | | | | | | |
| *An ASD of equal or more than 0.1 was considered to indicate a substantial imbalance between the two groups. | | | | | | | | | | | | | |
| †The rural area is constituted 19 prefectures with population of less than 200 inhabitants per km^2^. | | | | | | | | | | | | | |

**Table S3.** Baseline characteristics of unmatched and matched patients aged 40–49 years

| Characteristic | | | Pre-propensity-matched patients | | | | |  | Post-propensity-matched patients | | | | |
| --- | --- | --- | --- | --- | --- | --- | --- | --- | --- | --- | --- | --- | --- |
|  | | | Men | | Women | | ASD* |  | Men | | Women | | ASD* |
|  |  |  | (n=9110) | | (n=3845) | |  |  | (n=3840) | | (n=3840) | |  |
| Year | | |  |  |  |  |  |  |  |  |  |  |  |
|  | 2013 | | 2272 | (24.9) | 937 | (24.4) | 0.01 |  | 967 | (25.2) | 937 | (24.4) | 0.02 |
|  | 2014 | | 2211 | (24.3) | 944 | (24.6) | 0.01 |  | 932 | (24.3) | 941 | (24.5) | 0.01 |
|  | 2015 | | 2288 | (25.1) | 982 | (25.5) | 0.01 |  | 974 | (25.4) | 981 | (25.6) | <0.01 |
|  | 2016 | | 2339 | (25.7) | 982 | (25.5) | <0.01 |  | 967 | (25.2) | 981 | (25.6) | 0.01 |
| Geographic Japanese regions | | |  |  |  |  |  |  |  |  |  |  |  |
|  | Rural area† | | 1950 | (21.4) | 820 | (21.3) | <0.01 |  | 812 | (21.2) | 819 | (21.3) | <0.01 |
| Age, y, mean (SD) | | | 45.0 | (2.8) | 44.9 | (2.8) | 0.02 |  | 45.0 | (2.8) | 44.9 | (2.8) | 0.01 |
| Etiology of cardiac arrest | | |  |  |  |  |  |  |  |  |  |  |  |
|  | Presumed cardiac origin | | 6428 | (70.6) | 2204 | (57.3) | 0.28 |  | 2204 | (57.4) | 2201 | (57.3) | <0.01 |
| Initial cardiac rhythm | | |  |  |  |  |  |  |  |  |  |  |  |
|  | Shockable | | 2178 | (23.9) | 421 | (11.0) | 0.35 |  | 411 | (10.7) | 420 | (10.9) | 0.01 |
| Bystander witness status | | |  |  |  |  |  |  |  |  |  |  |  |
|  | No witness | | 5144 | (56.5) | 2328 | (60.6) | 0.08 |  | 2360 | (61.5) | 2326 | (60.6) | 0.02 |
|  | Witnessed by family member | | 2074 | (22.8) | 1060 | (27.6) | 0.11 |  | 1032 | (26.9) | 1058 | (27.6) | 0.02 |
|  | Witnessed by nonfamily member | | 1892 | (20.8) | 457 | (11.9) | 0.24 |  | 448 | (11.7) | 456 | (11.9) | 0.01 |
| Dispatcher CPR instruction | | |  |  |  |  |  |  |  |  |  |  |  |
|  | Offered | | 4918 | (54.0) | 2229 | (58.0) | 0.08 |  | 2201 | (57.3) | 2226 | (58.0) | 0.01 |
| Bystander intervention | | |  |  |  |  |  |  |  |  |  |  |  |
|  | Bystander defibrillation | | 9 | (0.1) | 1 | (0.03) | 0.03 |  | 0 | (0) | 1 | (0.03) | 0.02 |
|  | Bystander conventional CPR | | 581 | (6.4) | 265 | (6.9) | 0.02 |  | 258 | (6.7) | 265 | (6.9) | 0.01 |
|  | Bystander compression-only CPR | | 4047 | (44.4) | 1603 | (41.7) | 0.06 |  | 1587 | (41.3) | 1601 | (41.7) | 0.01 |
|  | Bystander rescue breathing-only CPR | | 17 | (0.2) | 13 | (0.3) | 0.03 |  | 11 | (0.3) | 12 | (0.3) | <0.01 |
|  | No bystander intervention | | 4456 | (48.9) | 1963 | (51.1) | 0.04 |  | 1984 | (51.7) | 1961 | (51.1) | 0.01 |
| Use of advanced airway management | | | 3860 | (42.4) | 1518 | (39.5) | 0.06 |  | 1499 | (39.0) | 1516 | (39.5) | 0.01 |
| Epinephrine administration | | | 2044 | (22.4) | 680 | (17.7) | 0.12 |  | 655 | (17.1) | 677 | (17.6) | 0.02 |
| Call-to-response time, min, mean (SD) | | | 9.25 | (4.1) | 9.17 | (3.7) | 0.02 |  | 9.20 | (3.8) | 9.17 | (3.7) | 0.01 |
| Call-to-hospital arrival time, min, mean (SD) | | | 31.8 | (10.6) | 32.4 | (10.1) | 0.05 |  | 32.4 | (10.5) | 32.4 | (10.1) | <0.01 |
| Values are reported as n (%) unless indicated otherwise. ASD, absolute standardized difference; CPR, cardiopulmonary resuscitation; SD, standard deviation. | | | | | | | | | | | | | |
| *An ASD of equal or more than 0.1 was considered to indicate a substantial imbalance between the two groups. | | | | | | | | | | | | | |
| †The rural area is constituted 19 prefectures with population of less than 200 inhabitants per km^2^. | | | | | | | | | | | | | |

**Table S4.** Baseline characteristics of unmatched and matched patients aged 50–59 years

| Characteristic | | | Pre-propensity-matched patients | | | | |  | Post-propensity-matched patients | | | | |
| --- | --- | --- | --- | --- | --- | --- | --- | --- | --- | --- | --- | --- | --- |
|  | | | Men | | Women | | ASD* |  | Men | | Women | | ASD* |
|  |  |  | (n=15,937) | | (n=5994) | |  |  | (n=5989) | | (n=5989) | |  |
| Year | | |  |  |  |  |  |  |  |  |  |  |  |
|  | 2013 | | 3958 | (24.8) | 1561 | (26.0) | 0.03 |  | 1554 | (26.0) | 1561 | (26.1) | <0.01 |
|  | 2014 | | 4010 | (25.2) | 1489 | (24.8) | 0.01 |  | 1468 | (24.5) | 1487 | (24.8) | 0.01 |
|  | 2015 | | 3990 | (25.0) | 1473 | (24.6) | 0.01 |  | 1487 | (24.8) | 1471 | (24.6) | 0.01 |
|  | 2016 | | 3979 | (25.0) | 1471 | (24.5) | 0.01 |  | 1480 | (24.7) | 1470 | (24.5) | <0.01 |
| Geographic Japanese regions | | |  |  |  |  |  |  |  |  |  |  |  |
|  | Rural area† | | 3903 | (24.5) | 1489 | (24.8) | 0.01 |  | 1507 | (25.2) | 1488 | (24.9) | 0.01 |
| Age, y, mean (SD) | | | 54.9 | (2.9) | 55 | (2.9) | 0.01 |  | 54.9 | (2.9) | 54.9 | (2.9) | <0.01 |
| Etiology of cardiac arrest | | |  |  |  |  |  |  |  |  |  |  |  |
|  | Presumed cardiac origin | | 11496 | (72.1) | 3427 | (57.2) | 0.32 |  | 3447 | (57.6) | 3424 | (57.2) | 0.01 |
| Initial cardiac rhythm | | |  |  |  |  |  |  |  |  |  |  |  |
|  | Shockable | | 3559 | (22.3) | 569 | (9.5) | 0.36 |  | 524 | (8.8) | 567 | (9.5) | 0.02 |
| Bystander witness status | | |  |  |  |  |  |  |  |  |  |  |  |
|  | No witness | | 9018 | (56.6) | 3805 | (63.5) | 0.14 |  | 3831 | (64.0) | 3802 | (63.5) | 0.01 |
|  | Witnessed by family member | | 3631 | (22.8) | 1595 | (26.6) | 0.09 |  | 1561 | (26.1) | 1594 | (26.6) | 0.01 |
|  | Witnessed by nonfamily member | | 3288 | (20.6) | 594 | (9.9) | 0.30 |  | 597 | (10.0) | 593 | (9.9) | <0.01 |
| Dispatcher CPR instruction | | |  |  |  |  |  |  |  |  |  |  |  |
|  | Offered | | 8571 | (53.8) | 3603 | (60.1) | 0.13 |  | 3608 | (60.2) | 3600 | (60.1) | <0.01 |
| Bystander intervention | | |  |  |  |  |  |  |  |  |  |  |  |
|  | Bystander defibrillation | | 17 | (0.1) | 1 | (0.02) | 0.04 |  | 1 | (0.02) | 0 | (0.0) | 0.02 |
|  | Bystander conventional CPR | | 1052 | (6.6) | 389 | (6.5) | <0.01 |  | 388 | (6.5) | 389 | (6.5) | <0.01 |
|  | Bystander compression-only CPR | | 6737 | (42.3) | 2620 | (43.7) | 0.03 |  | 2618 | (43.7) | 2620 | (43.8) | <0.01 |
|  | Bystander rescue breathing-only CPR | | 30 | (0.2) | 26 | (0.4) | 0.04 |  | 19 | (0.3) | 24 | (0.4) | 0.01 |
|  | No bystander intervention | | 8101 | (50.8) | 2958 | (49.4) | 0.03 |  | 2963 | (49.5) | 2956 | (49.4) | <0.01 |
| Use of advanced airway management | | | 6686 | (42.0) | 2403 | (40.1) | 0.04 |  | 2412 | (40.2) | 2401 | (40.1) | <0.01 |
| Epinephrine administration | | | 3604 | (22.6) | 1055 | (17.6) | 0.13 |  | 1068 | (17.8) | 1053 | (17.6) | 0.01 |
| Call-to-response time, min, mean (SD) | | | 9.36 | (4.3) | 9.19 | (3.8) | 0.04 |  | 9.21 | (3.8) | 9.19 | (3.8) | <0.01 |
| Call-to-hospital arrival time, min, mean (SD) | | | 31.7 | (10.6) | 32.1 | (10.3) | 0.03 |  | 32.0 | (10.4) | 32.1 | (10.3) | 0.01 |
| Values are reported as n (%) unless indicated otherwise. ASD, absolute standardized difference; CPR, cardiopulmonary resuscitation; SD, standard deviation. | | | | | | | | | | | | | |
| *An ASD of equal or more than 0.1 was considered to indicate a substantial imbalance between the two groups. | | | | | | | | | | | | | |
| †The rural area is constituted 19 prefectures with population of less than 200 inhabitants per km^2^. | | | | | | | | | | | | | |

**Table S5.** Baseline characteristics of unmatched and matched patients aged 60–69 years

| Characteristic | | | Pre-propensity-matched patients | | | | |  | Post-propensity-matched patients | | | | |
| --- | --- | --- | --- | --- | --- | --- | --- | --- | --- | --- | --- | --- | --- |
|  | | | Men | | Women | | ASD* |  | Men | | Women | | ASD* |
|  |  |  | (n=35,526) | | (n=14,857) | |  |  | (n=14,847) | | (n=14,847) | |  |
| Year | | |  |  |  |  |  |  |  |  |  |  |  |
|  | 2013 | | 8845 | (24.9) | 3698 | (24.9) | <0.01 |  | 3671 | (24.7) | 3698 | (24.9) | <0.01 |
|  | 2014 | | 9159 | (25.8) | 3774 | (25.4) | 0.01 |  | 3774 | (25.4) | 3773 | (25.4) | <0.01 |
|  | 2015 | | 8679 | (24.4) | 3698 | (24.9) | 0.01 |  | 3722 | (25.1) | 3694 | (24.9) | <0.01 |
|  | 2016 | | 8843 | (24.9) | 3687 | (24.8) | <0.01 |  | 3680 | (24.8) | 3682 | (24.8) | <0.01 |
| Geographic Japanese regions | | |  |  |  |  |  |  |  |  |  |  |  |
|  | Rural area† | | 8484 | (23.9) | 3601 | (24.2) | 0.01 |  | 3566 | (24.0) | 3599 | (24.2) | 0.01 |
| Age, y, mean (SD) | | | 65.0 | (2.8) | 65.0 | (2.7) | 0.08 |  | 65.2 | (2.7) | 65.2 | (2.7) | 0.01 |
| Etiology of cardiac arrest | | |  |  |  |  |  |  |  |  |  |  |  |
|  | Presumed cardiac origin | | 25706 | (72.4) | 9561 | (64.4) | 0.17 |  | 9649 | (65.0) | 9555 | (64.4) | 0.01 |
| Initial cardiac rhythm | | |  |  |  |  |  |  |  |  |  |  |  |
|  | Shockable | | 6172 | (17.4) | 1084 | (7.3) | 0.31 |  | 1057 | (7.1) | 1083 | (7.3) | 0.01 |
| Bystander witness status | | |  |  |  |  |  |  |  |  |  |  |  |
|  | No witness | | 20440 | (57.5) | 9288 | (62.5) | 0.10 |  | 9395 | (63.3) | 9283 | (62.5) | 0.02 |
|  | Witnessed by family member | | 9245 | (26.0) | 4154 | (28.0) | 0.04 |  | 4066 | (27.4) | 4150 | (28.0) | 0.01 |
|  | Witnessed by nonfamily member | | 5841 | (16.4) | 1415 | (9.5) | 0.21 |  | 1386 | (9.3) | 1414 | (9.5) | 0.01 |
| Dispatcher CPR instruction | | |  |  |  |  |  |  |  |  |  |  |  |
|  | Offered | | 19637 | (55.3) | 8935 | (60.1) | 0.10 |  | 8938 | (60.2) | 8931 | (60.2) | <0.01 |
| Bystander intervention | | |  |  |  |  |  |  |  |  |  |  |  |
|  | Bystander defibrillation | | 31 | (0.1) | 2 | (0.01) | 0.03 |  | 2 | (0.01) | 2 | (0.01) | 0 |
|  | Bystander conventional CPR | | 1963 | (5.5) | 826 | (5.6) | <0.01 |  | 817 | (5.5) | 824 | (5.6) | <0.01 |
|  | Bystander compression-only CPR | | 14968 | (42.1) | 6571 | (44.2) | 0.04 |  | 6514 | (43.9) | 6569 | (44.2) | 0.01 |
|  | Bystander rescue breathing-only CPR | | 76 | (0.2) | 40 | (0.3) | 0.01 |  | 45 | (0.3) | 40 | (0.3) | 0.01 |
|  | No bystander intervention | | 18488 | (52.0) | 7418 | (49.9) | 0.04 |  | 7469 | (50.3) | 7412 | (49.9) | 0.01 |
| Use of advanced airway management | | | 15748 | (44.3) | 6474 | (43.6) | 0.02 |  | 6456 | (43.5) | 6468 | (43.6) | <0.01 |
| Epinephrine administration | | | 7829 | (22.0) | 2826 | (19.0) | 0.07 |  | 2775 | (18.7) | 2825 | (19.0) | 0.01 |
| Call-to-response time, min, mean (SD) | | | 9.32 | (4.2) | 9.14 | (3.8) | 0.04 |  | 9.18 | (3.9) | 9.14 | (3.8) | 0.01 |
| Call-to-hospital arrival time, min, mean (SD) | | | 31.7 | (10.6) | 31.6 | (10.1) | <0.01 |  | 31.6 | (10.5) | 31.6 | (10.1) | <0.01 |
| Values are reported as n (%) unless indicated otherwise. ASD, absolute standardized difference; CPR, cardiopulmonary resuscitation; SD, standard deviation. | | | | | | | | | | | | | |
| *An ASD of equal or more than 0.1 was considered to indicate a substantial imbalance between the two groups. | | | | | | | | | | | | | |
| †The rural area is constituted 19 prefectures with population of less than 200 inhabitants per km^2^. | | | | | | | | | | | | | |

**Table S6.** Baseline characteristics of unmatched and matched patients aged 70–79 years

| Characteristic | | | Pre-propensity-matched patients | | | | |  | Post-propensity-matched patients | | | | |
| --- | --- | --- | --- | --- | --- | --- | --- | --- | --- | --- | --- | --- | --- |
|  | | | Men | | Women | | ASD* |  | Men | | Women | | ASD* |
|  |  |  | (n=57,386) | | (n=32,294) | |  |  | (n=32,260) | | (n=32,260) | |  |
| Year | | |  |  |  |  |  |  |  |  |  |  |  |
|  | 2013 | | 14,190 | (24.7) | 7914 | (24.5) | 0.01 |  | 7873 | (24.4) | 7906 | (24.5) | <0.01 |
|  | 2014 | | 14,899 | (26.0) | 8359 | (25.9) | <0.01 |  | 8361 | (25.9) | 8346 | (25.9) | <0.01 |
|  | 2015 | | 14,263 | (24.9) | 8059 | (25.0) | <0.01 |  | 8094 | (25.1) | 8053 | (25.0) | <0.01 |
|  | 2016 | | 14,034 | (24.5) | 7962 | (24.7) | <0.01 |  | 7932 | (24.6) | 7955 | (24.7) | <0.01 |
| Geographic Japanese regions | | |  |  |  |  |  |  |  |  |  |  |  |
|  | Rural area† | | 13,328 | (23.2) | 7452 | (23.1) | <0.01 |  | 7378 | (22.9) | 7444 | (23.1) | <0.01 |
| Age, y, mean (SD) | | | 74.9 | (2.9) | 75.0 | (2.8) | 0.11 |  | 75.2 | (2.8) | 75.2 | (2.8) | <0.01 |
| Etiology of cardiac arrest | | |  |  |  |  |  |  |  |  |  |  |  |
|  | Presumed cardiac origin | | 40,198 | (70.1) | 22,253 | (68.9) | 0.02 |  | 22,156 | (68.7) | 22,231 | (68.9) | 0.01 |
| Initial cardiac rhythm | | |  |  |  |  |  |  |  |  |  |  |  |
|  | Shockable | | 5780 | (10.1) | 1747 | (5.4) | 0.18 |  | 1780 | (5.5) | 1747 | (5.4) | <0.01 |
| Bystander witness status | | |  |  |  |  |  |  |  |  |  |  |  |
|  | No witness | | 34,023 | (59.3) | 20,902 | (64.7) | 0.11 |  | 20,954 | (65.0) | 20,881 | (64.7) | <0.01 |
|  | Witnessed by family member | | 16,887 | (29.4) | 7810 | (24.2) | 0.12 |  | 7809 | (24.2) | 7802 | (24.2) | <0.01 |
|  | Witnessed by nonfamily member | | 6476 | (11.3) | 3582 | (11.1) | 0.01 |  | 3497 | (10.8) | 3577 | (11.1) | 0.01 |
| Dispatcher CPR instruction | | |  |  |  |  |  |  |  |  |  |  |  |
|  | Offered | | 33,485 | (58.4) | 19,856 | (61.5) | 0.06 |  | 19,947 | (61.8) | 19,840 | (61.5) | 0.01 |
| Bystander intervention | | |  |  |  |  |  |  |  |  |  |  |  |
|  | Bystander defibrillation | | 23 | (0.04) | 6 | (0.02) | 0.01 |  | 8 | (0.02) | 6 | (0.02) | <0.01 |
|  | Bystander conventional CPR | | 2656 | (4.6) | 1921 | (6.0) | 0.06 |  | 1918 | (6.0) | 1919 | (6.0) | <0.01 |
|  | Bystander compression-only CPR | | 24,055 | (41.9) | 14,508 | (44.9) | 0.06 |  | 14,601 | (45.3) | 14,497 | (44.9) | 0.01 |
|  | Bystander rescue breathing-only CPR | | 123 | (0.2) | 76 | (0.2) | <0.01 |  | 65 | (0.2) | 76 | (0.2) | 0.01 |
|  | No bystander intervention | | 30,529 | (53.2) | 15,783 | (48.9) | 0.09 |  | 15,668 | (48.6) | 15,762 | (48.9) | 0.01 |
| Use of advanced airway management | | | 26,052 | (45.4) | 14,343 | (44.4) | 0.02 |  | 14,317 | (44.4) | 14,332 | (44.4) | <0.01 |
| Epinephrine administration | | | 12,161 | (21.2) | 5958 | (18.5) | 0.07 |  | 6041 | (18.7) | 5954 | (18.5) | 0.01 |
| Call-to-response time, min, mean (SD) | | | 9.14 | (3.8) | 9.05 | (3.6) | 0.02 |  | 9.03 | (3.7) | 9.05 | (3.6) | <0.01 |
| Call-to-hospital arrival time, min, mean (SD) | | | 31.9 | (10.2) | 31.6 | (10.1) | 0.02 |  | 31.6 | (10.1) | 31.6 | (10.1) | 0.01 |
| Values are reported as n (%) unless indicated otherwise. ASD, absolute standardized difference; CPR, cardiopulmonary resuscitation; SD, standard deviation. | | | | | | | | | | | | | |
| *An ASD of equal or more than 0.1 was considered to indicate a substantial imbalance between the two groups. | | | | | | | | | | | | | |
| †The rural area is constituted 19 prefectures with population of less than 200 inhabitants per km^2^. | | | | | | | | | | | | | |

**Table S7.** Baseline characteristics of unmatched and matched patients aged 80–89 years

| Characteristic | | | Pre-propensity-matched patients | | | | |  | Post-propensity-matched patients | | | | |
| --- | --- | --- | --- | --- | --- | --- | --- | --- | --- | --- | --- | --- | --- |
|  | | | Men | | Women | | ASD* |  | Men | | Women | | ASD* |
|  |  |  | (n=73,303) | | (n=66,004) | |  |  | (n=59,734) | | (n=59,734) | |  |
| Year | | |  |  |  |  |  |  |  |  |  |  |  |
|  | 2013 | | 18,149 | (24.8) | 16475 | (25.0) | <0.01 |  | 14,826 | (24.8) | 14,835 | (24.8) | <0.01 |
|  | 2014 | | 18,428 | (25.1) | 16753 | (25.4) | 0.01 |  | 15,126 | (25.3) | 15,149 | (25.4) | <0.01 |
|  | 2015 | | 18,363 | (25.1) | 16460 | (24.9) | <0.01 |  | 14,976 | (25.1) | 14,906 | (25.0) | <0.01 |
|  | 2016 | | 18,363 | (25.1) | 16316 | (24.7) | 0.01 |  | 14,806 | (24.8) | 14,844 | (24.9) | <0.01 |
| Geographic Japanese regions | | |  |  |  |  |  |  |  |  |  |  |  |
|  | Rural area† | | 19,566 | (26.7) | 17598 | (26.7) | <0.01 |  | 15,982 | (26.8) | 15,961 | (26.7) | <0.01 |
| Age, y, mean (SD) | | | 84.3 | (2.8) | 85.0 | (2.8) | 0.20 |  | 84.6 | (2.8) | 84.6 | (2.8) | <0.01 |
| Etiology of cardiac arrest | | |  |  |  |  |  |  |  |  |  |  |  |
|  | Presumed cardiac origin | | 51,048 | (69.6) | 47,059 | (71.3) | 0.04 |  | 42,431 | (71.0) | 42,381 | (71.0) | <0.01 |
| Initial cardiac rhythm | | |  |  |  |  |  |  |  |  |  |  |  |
|  | Shockable | | 3568 | (4.9) | 2182 | (3.3) | 0.08 |  | 2146 | (3.6) | 2136 | (3.6) | <0.01 |
| Bystander witness status | | |  |  |  |  |  |  |  |  |  |  |  |
|  | No witness | | 45,066 | (61.5) | 42,808 | (64.9) | 0.07 |  | 39,342 | (65.9) | 39,395 | (66.0) | <0.01 |
|  | Witnessed by family member | | 20,290 | (27.7) | 12654 | (19.2) | 0.20 |  | 12,666 | (21.2) | 12,616 | (21.1) | <0.01 |
|  | Witnessed by nonfamily member | | 7947 | (10.8) | 10542 | (16.0) | 0.15 |  | 7726 | (12.9) | 7723 | (12.9) | <0.01 |
| Dispatcher CPR instruction | | |  |  |  |  |  |  |  |  |  |  |  |
|  | Offered | | 44,444 | (60.6) | 41,562 | (63.0) | 0.05 |  | 37,344 | (62.5) | 37,374 | (62.6) | <0.01 |
| Bystander intervention | | |  |  |  |  |  |  |  |  |  |  |  |
|  | Bystander defibrillation | | 22 | (0.03) | 15 | (0.02) | <0.01 |  | 11 | (0.02) | 15 | (0.03) | <0.01 |
|  | Bystander conventional CPR | | 3987 | (5.4) | 5401 | (8.2) | 0.11 |  | 3846 | (6.4) | 3882 | (6.5) | <0.01 |
|  | Bystander compression-only CPR | | 32,563 | (44.4) | 32,528 | (49.3) | 0.10 |  | 28,536 | (47.8) | 28,564 | (47.8) | <0.01 |
|  | Bystander rescue breathing-only CPR | | 167 | (0.2) | 190 | (0.3) | 0.01 |  | 153 | (0.3) | 151 | (0.3) | <0.01 |
|  | No bystander intervention | | 36,564 | (49.9) | 27,870 | (42.2) | 0.15 |  | 27,188 | (45.5) | 27,122 | (45.4) | <0.01 |
| Use of advanced airway management | | | 32,055 | (43.7) | 27,659 | (41.9) | 0.04 |  | 25,338 | (42.4) | 25,397 | (42.5) | <0.01 |
| Epinephrine administration | | | 14,474 | (19.8) | 11,171 | (16.9) | 0.07 |  | 10,454 | (17.5) | 10,477 | (17.5) | <0.01 |
| Call-to-response time, min, mean (SD) | | | 9.24 | (3.8) | 9.12 | (3.6) | 0.03 |  | 9.16 | (3.7) | 9.16 | (3.7) | <0.01 |
| Call-to-hospital arrival time, min, mean (SD) | | | 31.8 | (10.3) | 31.5 | (10.1) | 0.03 |  | 31.6 | (10.2) | 31.6 | (10.1) | <0.01 |
| Values are reported as n (%) unless indicated otherwise. ASD, absolute standardized difference; CPR, cardiopulmonary resuscitation; SD, standard deviation. | | | | | | | | | | | | | |
| *An ASD of equal or more than 0.1 was considered to indicate a substantial imbalance between the two groups. | | | | | | | | | | | | | |
| †The rural area is constituted 19 prefectures with population of less than 200 inhabitants per km^2^. | | | | | | | | | | | | | |

**Table S8.** Baseline characteristics of unmatched and matched patients aged ≥90 years

| Characteristic | | | Pre-propensity-matched patients | | | | |  | Post-propensity-matched patients | | | | |
| --- | --- | --- | --- | --- | --- | --- | --- | --- | --- | --- | --- | --- | --- |
|  | | | Men | | Women | | ASD* |  | Men | | Women | | ASD* |
|  |  |  | (n=20,476) | | (n=43,943) | |  |  | (n=20,453) | | (n=20,453) | |  |
| Year | | |  |  |  |  |  |  |  |  |  |  |  |
|  | 2013 | | 4,637 | (22.7) | 10506 | (23.9) | 0.03 |  | 4,635 | (22.7) | 4,658 | (22.8) | <0.01 |
|  | 2014 | | 5,047 | (24.7) | 10965 | (25.0) | 0.01 |  | 5,039 | (24.6) | 5,057 | (24.7) | <0.01 |
|  | 2015 | | 5,123 | (25.0) | 11026 | (25.1) | <0.01 |  | 5,116 | (25.0) | 5,089 | (24.9) | <0.01 |
|  | 2016 | | 5,669 | (27.7) | 11446 | (26.1) | 0.04 |  | 5,663 | (27.7) | 5,649 | (27.6) | <0.01 |
| Geographic Japanese regions | | |  |  |  |  |  |  |  |  |  |  |  |
|  | Rural area† | | 5,614 | (27.4) | 11981 | (27.3) | <0.01 |  | 5,608 | (27.4) | 5,567 | (27.2) | <0.01 |
| Age, y, mean (SD) | | | 92.8 | (2.8) | 94 | (3.2) | 0.29 |  | 92.8 | (2.8) | 92.8 | (2.8) | <0.01 |
| Etiology of cardiac arrest | | |  |  |  |  |  |  |  |  |  |  |  |
|  | Presumed cardiac origin | | 14,344 | (70.1) | 31,284 | (71.2) | 0.03 |  | 14,327 | (70.1) | 14,306 | (70.0) | <0.01 |
| Initial cardiac rhythm | | |  |  |  |  |  |  |  |  |  |  |  |
|  | Shockable | | 643 | (3.1) | 975 | (2.2) | 0.06 |  | 640 | (3.1) | 635 | (3.1) | <0.01 |
| Bystander witness status | | |  |  |  |  |  |  |  |  |  |  |  |
|  | No witness | | 12,781 | (62.4) | 27,074 | (61.6) | 0.02 |  | 12,766 | (62.4) | 12,751 | (62.3) | <0.01 |
|  | Witnessed by family member | | 4,697 | (22.9) | 7732 | (17.6) | 0.13 |  | 4,689 | (22.9) | 4,742 | (23.2) | 0.01 |
|  | Witnessed by nonfamily member | | 2998 | (14.6) | 9137 | (20.8) | 0.16 |  | 2998 | (14.7) | 2960 | (14.5) | 0.01 |
| Dispatcher CPR instruction | | |  |  |  |  |  |  |  |  |  |  |  |
|  | Offered | | 12,624 | (61.7) | 27,404 | (62.4) | 0.01 |  | 12,613 | (61.7) | 12,578 | (61.5) | <0.01 |
| Bystander intervention | | |  |  |  |  |  |  |  |  |  |  |  |
|  | Bystander defibrillation | | 5 | (0.02) | 16 | (0.04) | 0.01 |  | 5 | (0.02) | 5 | (0.02) | 0 |
|  | Bystander conventional CPR | | 1489 | (7.3) | 4512 | (10.3) | 0.11 |  | 1486 | (7.3) | 1420 | (6.9) | 0.01 |
|  | Bystander compression-only CPR | | 10,009 | (48.9) | 22,632 | (51.5) | 0.05 |  | 10,003 | (48.9) | 10,007 | (48.9) | <0.01 |
|  | Bystander rescue breathing-only CPR | | 46 | (0.2) | 117 | (0.3) | 0.01 |  | 46 | (0.2) | 40 | (0.2) | 0.01 |
|  | No bystander intervention | | 8,927 | (43.6) | 16,666 | (37.9) | 0.12 |  | 8,913 | (43.6) | 8,981 | (43.9) | 0.01 |
| Use of advanced airway management | | | 8,033 | (39.2) | 15,867 | (36.1) | 0.06 |  | 8,023 | (39.2) | 7,926 | (38.8) | 0.01 |
| Epinephrine administration | | | 3,429 | (16.8) | 6,095 | (13.9) | 0.08 |  | 3,422 | (16.7) | 3,354 | (16.4) | 0.01 |
| Call-to-response time, min, mean (SD) | | | 9.19 | (3.7) | 9.09 | (3.5) | 0.03 |  | 9.19 | (3.7) | 9.18 | (3.6) | <0.01 |
| Call-to-hospital arrival time, min, mean (SD) | | | 31.4 | (10.1) | 31.1 | (9.9) | 0.03 |  | 31.4 | (10.0) | 31.3 | (9.9) | 0.01 |
| Values are reported as n (%) unless indicated otherwise. ASD, absolute standardized difference; CPR, cardiopulmonary resuscitation; SD, standard deviation. | | | | | | | | | | | | | |
| *An ASD of equal or more than 0.1 was considered to indicate a substantial imbalance between the two groups. | | | | | | | | | | | | | |
| †The rural area is constituted 19 prefectures with population of less than 200 inhabitants per km^2^. | | | | | | | | | | | | | |
