# Supplementary figures and images for: Sex-specific differences in survival after out-of-hospital cardiac arrest: a nationwide, population-based observational study
Source: Crit Care. 2019 Jul 25;23:263. doi: 10.1186/s13054-019-2547-x (PMC6659261; doi:10.1186/s13054-019-2547-x)

## Slide 1
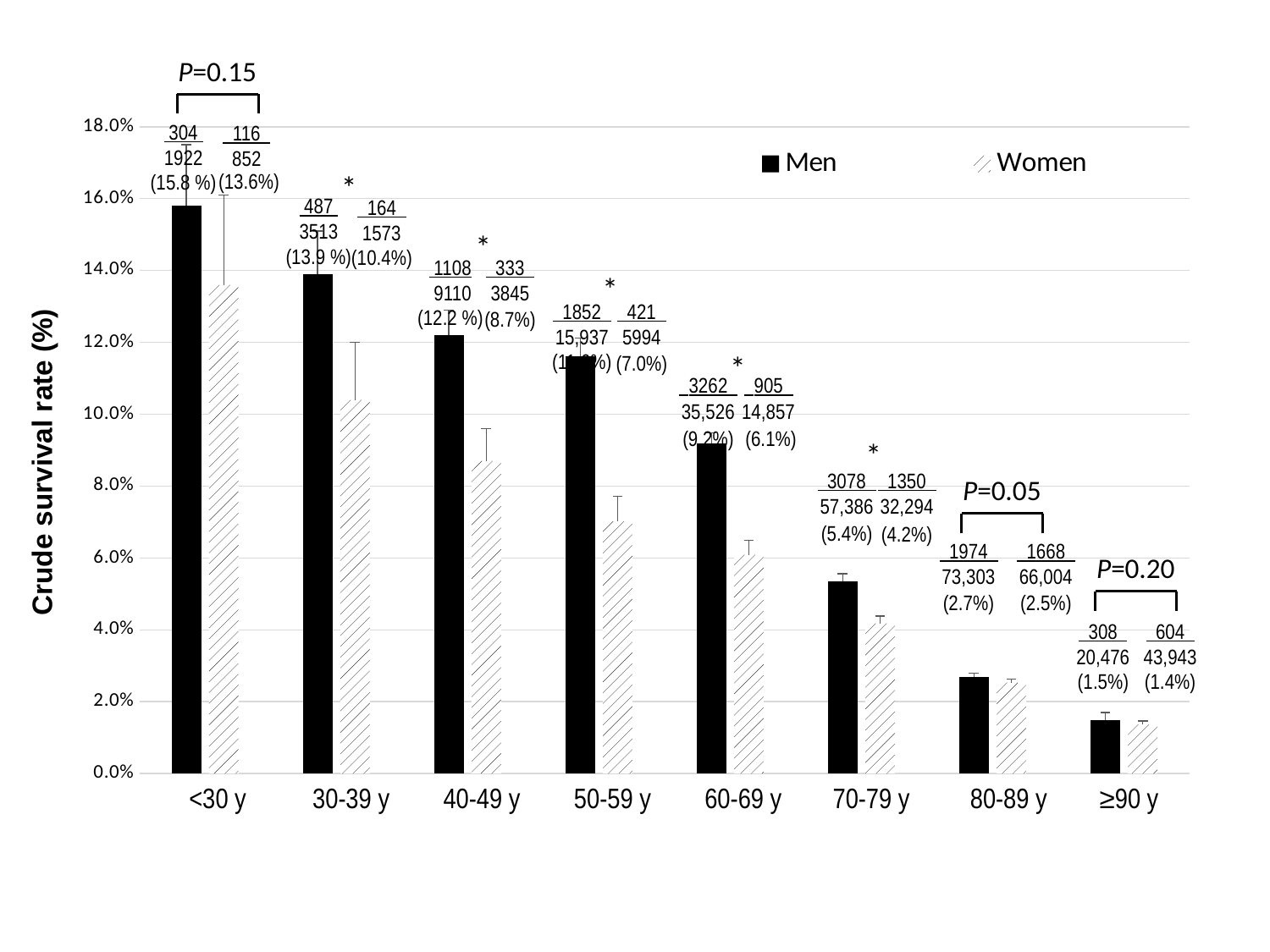

P=0.15
### Chart
| Category | Men | Women |
|---|---|---|
| <30 | 0.158 | 0.136 |
| 30-39 | 0.139 | 0.104 |
| 40-49 | 0.122 | 0.087 |
| 50-59 | 0.1162 | 0.0702 |
| 60-69 | 0.0918 | 0.0609 |
| 70-79 | 0.0536 | 0.0418 |
| 80-89 | 0.0269 | 0.0253 |
| >89 | 0.015 | 0.0137 | 304
1922
(15.8 %)
 116
852
(13.6%)
Crude survival rate (%)
*
 487
3513
(13.9 %)
 164
1573
(10.4%)
*
 1108
 9110
(12.2 %)
 333
3845
(8.7%)
*
 1852
15,937
(11.6%)
 421
5994
(7.0%)
*
 3262
35,526
(9.2%)
 905
14,857
(6.1%)
*
 3078
57,386
(5.4%)
 1350
32,294
(4.2%)
P=0.05
 1974
73,303
(2.7%)
 1668
66,004
(2.5%)
P=0.20
 308
20,476
(1.5%)
 604
43,943
(1.4%)
<30 y
30-39 y
40-49 y
50-59 y
60-69 y
70-79 y
80-89 y
≥90 y

Supplement: Supplementary file 2 — Figure S1. One-month crude survival rate in unmatched patients by age *P < 0.001. The trends of both sexes for increasing age groups were significant (all P for trend < 0.001). (PPTX 503 kb) [file 13054_2019_2547_MOESM2_ESM.pptx]

## Slide 1
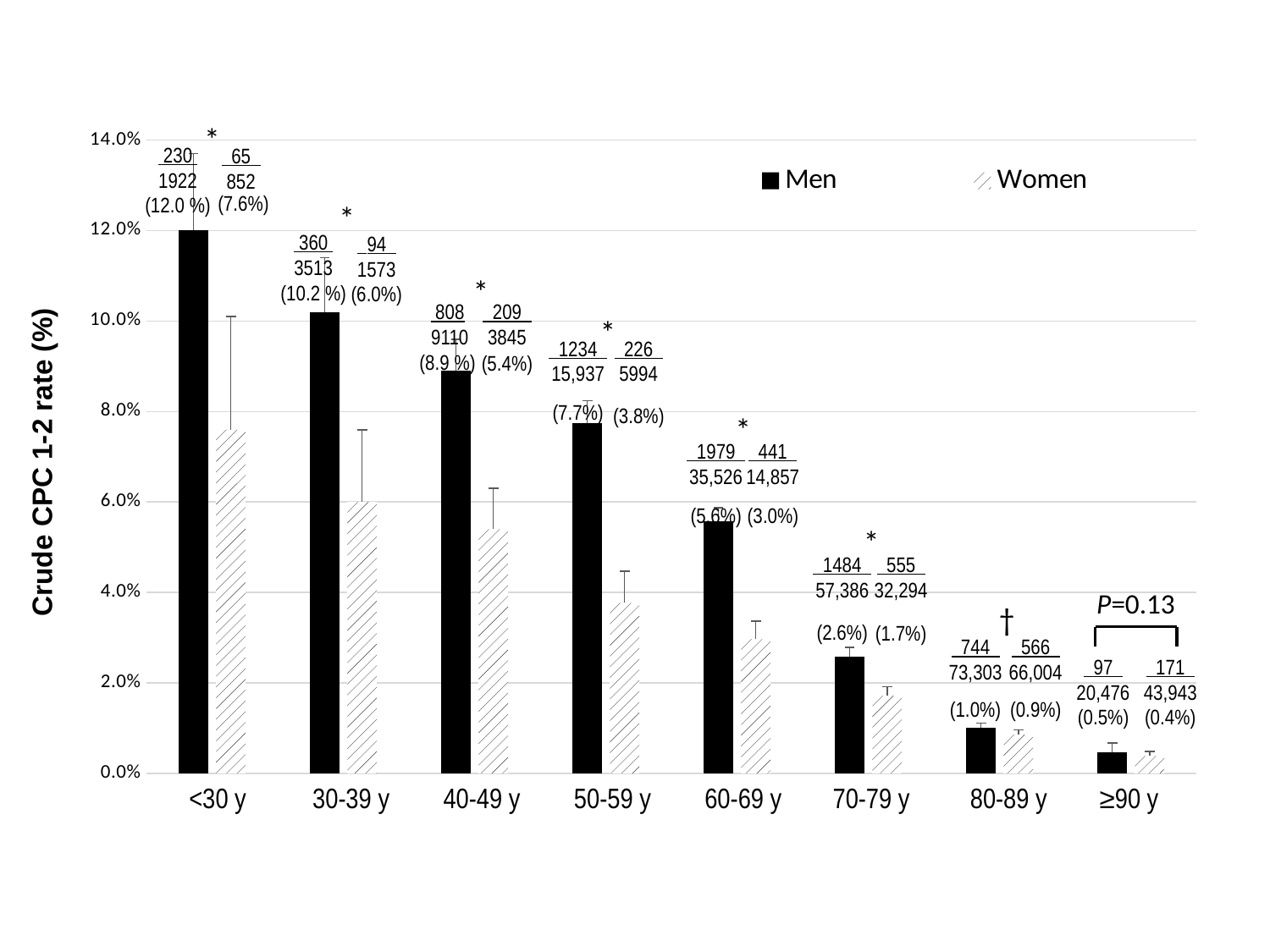

*
### Chart
| Category | Men | Women |
|---|---|---|
| <30 | 0.12 | 0.076 |
| 30-39 | 0.102 | 0.06 |
| 40-49 | 0.089 | 0.054 |
| 50-59 | 0.0774 | 0.0377 |
| 60-69 | 0.0557 | 0.0297 |
| 70-79 | 0.0259 | 0.0172 |
| 80-89 | 0.0101 | 0.0086 |
| >89 | 0.0047 | 0.0039 | 230
1922
(12.0 %)
 65
852
(7.6%)
Crude CPC 1-2 rate (%)
*
 360
3513
(10.2 %)
 94
1573
(6.0%)
*
 808
 9110
(8.9 %)
 209
3845
(5.4%)
*
 1234
15,937
(7.7%)
 226
5994
(3.8%)
*
 1979
35,526
(5.6%)
 441
14,857
(3.0%)
*
 1484
57,386
(2.6%)
 555
32,294
(1.7%)
P=0.13
†
 744
73,303
(1.0%)
 566
66,004
(0.9%)
 97
20,476
(0.5%)
 171
43,943
(0.4%)
<30 y
30-39 y
40-49 y
50-59 y
60-69 y
70-79 y
80-89 y
≥90 y

Supplement: Supplementary file 3 — Figure S2. One-month crude CPC 1–2 rate in unmatched patients by age CPC: Cerebral Performance Category scale. *P < 0.001. †P < 0.005. The trends of both sexes for increasing age groups were significant (all P for trend < 0.001). (PPTX 534 kb) [file 13054_2019_2547_MOESM3_ESM.pptx]
